# Plasma Leptin Levels, Obstructive Sleep Apnea Syndrome, and Diabetes Are Associated with Obesity-Related Alterations of Peripheral Blood Monocyte Subsets

**DOI:** 10.4049/immunohorizons.2300009

**Published:** 2023-03-15

**Authors:** Svenja Meyhöfer, Armin Steffen, Kirstin Plötze-Martin, Christian Lange, Jens-Uwe Marquardt, Karl-Ludwig Bruchhage, Sebastian M. Meyhöfer, Ralph Pries

**Affiliations:** * Department of Medicine 1, University Hospital of Schleswig-Holstein, Lübeck, Germany; † Institute for Endocrinology & Diabetes, Department of Internal Medicine 1, University Hospital of Schleswig-Holstein, Lübeck, Germany; ‡ Department of Otorhinolaryngology, University Hospital of Schleswig-Holstein, Lübeck, Germany; § German Center for Diabetes Research, Neuherberg, Germany

## Abstract

Obesity is a dramatically increasing disease, accompanied with comorbidities such as cardiovascular disease and obstructive sleep apnea syndrome (OSAS). Both obesity and OSAS per se are associated with systemic inflammation. However, the multifactorial impact of obesity, OSAS, and its concomitant diseases on the immunological characteristics of circulating monocytes has not yet been fully resolved. Monocyte subsets of 82 patients with obesity were analyzed in whole blood measurements in terms of the CD14/CD16 cell surface expression patterns and different monocytic adhesion molecules using flow cytometry. Plasma levels of adipokines adiponectin and leptin of all patients were evaluated and correlated with accompanying cellular and clinical values. Whole blood measurements revealed a significant overall redistribution of CD14/CD16 monocyte subsets in patients with obesity. Monocytic adhesion molecules CD11a, CD11b, and CX3CR1 were significantly elevated. The observed alterations significantly correlated with plasma leptin levels and diabetes status as crucial amplifying factors. The additive impact of obesity, diabetes, and OSAS on the immunological balance of peripheral blood monocytes requires a coordinated regimen in terms of therapeutic treatment, respiratory support, and weight loss to improve the systemic immunity in these patients.

## Introduction

The prevalence of obesity is dramatically increasing worldwide and has more than doubled since 1980 (1, 2). Obesity is characterized by an increased abnormal accumulation of body fat and accompanied with comorbid diseases, such as diabetes mellitus, cardiovascular disease, atherosclerosis, and cancer, respectively (3–5). Furthermore, obesity is associated with systemic inflammation and immune cell recruitment to metabolic tissues (6, 7). The autophagic activity of PBMCs is known to correlate as well with obesity-related body fat percentage (8). In adipose tissue, increased abundances of proinflammatory immune cells such as M1 macrophages and CD8^+^ T cells have been observed, which secrete proinflammatory cytokines such as IL-1β, IL-6, IL-17, and IFN-γ (9). From a clinical point of view, it is well known that obesity is a major risk factor for obstructive sleep apnea syndrome (OSAS) (10), a disease associated with subclinical inflammation.

Different studies have linked the development or worsening of OSAS to increasing weight (11). The synergistic effect of obesity and OSAS was shown to increase the incidence of metabolic diseases, such as dyslipidemia, hypertension, insulin resistance, cardiovascular diseases, and nonalcoholic fatty liver disease (12).

We have recently shown that OSAS patients reveal a redistribution of monocyte subsets and subsequently an imbalanced PD-1/PD-L1 communication with CD4/CD8 T cells (13).

In general, monocytes can be subdivided in view of their CD14 and CD16 surface expression levels. The major population (85–90%) of human monocytes is CD14^2+^CD16^−^ “classical” monocytes (CMs). They are classified as “naive-like” monocytes that arise from hematopoietic stem cells in the bone marrow and are released into the bloodstream. The minor CD14^+^CD16^+^ “intermediate” (IM) and CD14^dim+^CD16^+^ “nonclassical” monocyte (NCM) subsets are considered to be more differentiated and proinflammatory monocytes that exert specialized functions, such as viral defense and patrolling behavior (14, 15). Under physiological conditions, they comprise each an amount of 5–10% of peripheral blood monocytes. An increase of these proinflammatory subsets has been shown in various inflammatory diseases, such as rheumatoid arthritis, asthma, and also obesity (13, 16–19). However, the complex impact of obesity, OSAS, and its concomitant diseases on the peripheral composition and immunological consequences of blood monocyte subsets has not really been separately considered yet.

This study investigates abundances and alterations of circulating CD14/CD16 monocyte subsets in patients with obesity with and without OSAS and on the background of manifold clinicopathological phenotypes. The three monocyte subtypes are known to differentially express various chemokine receptors in response to different environmental signals (20). Therefore, expression of adhesion molecules and chemokine receptors CD11a (integrin-α L; LFA-1), CD11b (integrin-α M; Mac-1), CD11c (integrin-α X), CD18 (integrin β-2), CD29 (integrin β-1), CD49d (integrin β-4), CD162 (P-selectin receptor), and CX3CR1 (CX3CL1 receptor) was analyzed on monocyte subsets using flow cytometry. Furthermore, plasma levels of adipokines adiponectin and leptin of all patients were evaluated and correlated with accompanying cellular and clinical values. The study aimed to better understand the context of obesity and its accompanying diseases, the individual situation of our patients, and the immunological balance and characteristics of peripheral blood monocyte subsets.

## Materials and Methods

### Ethics statement and blood collection

All patients were clinically examined at the Department of Internal Medicine 1, University Hospital Schleswig-Holstein, Campus Lübeck. The study was approved by the local ethics committee of the University of Lübeck (approval number 21-183) and conducted in accordance with the ethical principles for medical research formulated in the World Medical Association Declaration of Helsinki. All subjects have signed an informed written consent and were clarified about the aims of the study and the use of their samples. Blood samples were collected between January and September 2022 from healthy donors (*n* = 27) and patients with obesity (*n* = 82) with body mass index (BMI) >35.0 kg/m^2^. Blood was drawn by venipuncture into a sodium citrate containing S-Monovette (Sarstedt, Nümbrecht, Germany). The clinicopathological characteristics of the patients are listed in Table I.

### Staining of monocyte subsets in whole blood

Within 4 h after blood collection, 20 µl of citrate blood was diluted in 80 µl PBS. Blood cells were stained with the following Abs: CD45-PE, CD14-FITC, CD16-BV-510, HLA-DR-allophycocyanin-Cy7, CX3CR1-BV421, CD11a-PE-Cy7, CD11b-PerCP, CD11c-BV421, CD18-allophycocyanin, CD162-PE, CD29-PE-CY7, and CD49d-allophycocyanin (all from BioLegend, San Diego, CA). After 25-min staining in the dark, 650 µl RBC Lysis Buffer (BioLegend) was added to the samples and incubated for another 20 min. Subsequently, the suspension was centrifuged at 400 × *g* for 5 min, and supernatant was discarded. Cell pellet was resuspended in 100 µl fresh PBS and used for FACS analysis.

### FACS analysis

Flow cytometry was performed with a MACSQuant 10 flow cytometer (Miltenyi Biotec, Bergisch-Gladbach, Germany), and data were analyzed using FlowJo software version 10.0 (FlowJo, Ashland, OR). All Ab titrations and compensations were performed beforehand. For whole blood measurements, at least 100,000 CD45^+^ leukocytes were analyzed. Gating of monocyte subsets was performed as described previously (13).

### Adipokine analysis

Concentrations of adipokines adiponectin and leptin were assessed from EDTA-plasma samples and were determined by ELISAs according to the manufacturer’s protocols (R&D Systems, Minneapolis, MN).

### Statistical analysis

GraphPad Prism Version 7.0f (GraphPad Software, San Diego, CA) was used for unpaired Student *t* tests for statistical analysis of all data presented in this article. The mean and SEs (SEMs) are presented. The *p* values <0.05 were considered to be significant. Correlation analysis between different parameters was calculated using multivariate regression with the Pearson correlation coefficient; *p < 0.05, **p < 0.01, and ***p < 0.001. Additional statistical details are given in the respective figure legends when appropriate.

## Results

### Obesity-related distribution of peripheral monocyte subsets

In this study, we analyzed the abundances and characteristics of blood monocyte subsets of 82 patients with obesity (64 female) with a median age of 46 y and a median BMI of 46.8 kg/m^2^. Healthy control subjects (*n* = 27, 19 female) had a median age of 48 y and a median BMI of 24.6 kg/m^2^. Different clinical parameters were diagnosed, such as hypertension, diabetes mellitus, OSAS, nonalcoholic fatty liver disease, cholesterol, glycated hemoglobin (HbA1c) values, and/or lipid-lowering medications, respectively. The clinicopathological characteristics of all subjects are listed in Table I.

**Table I.** Clinicopathological parameters

	Patients (*n* = 82)	
Characteristics	*n*	%
Gender		
Male	18	22
Female	64	78
BMI		
≤50	57	69
>50	25	31
Diabetes		
Yes	32	39
No	50	61
Fatty liver		
Yes	73	89
No	2	3
N/A	7	8
Hypertension		
Yes	46	56
No	36	44
OSAS		
Yes	56	68
No	14	17
N/A	12	15
Tobacco consumption		
Yes	9	12
No	66	80
N/A	7	8

N/A, not available.

**FIGURE 1.**
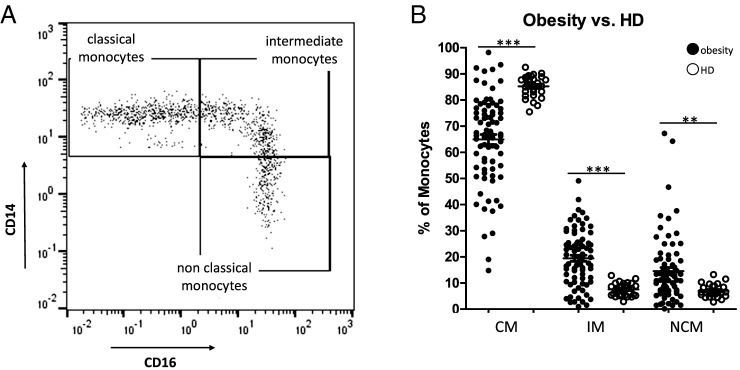
Flow cytometric analysis of CD14/CD16 characterized monocyte subsets. (**A**) Representative example gating scheme of peripheral monocyte subset analysis by flow cytometry. (**B**) Whole blood analysis revealed significantly decreased abundances of CMs accompanied by significantly increased percentages of IMs and NCMs in patients with obesity (*n* = 82) compared with healthy donors (*n* = 27). **p < 0.01, ***p < 0.001.

**FIGURE 2.**
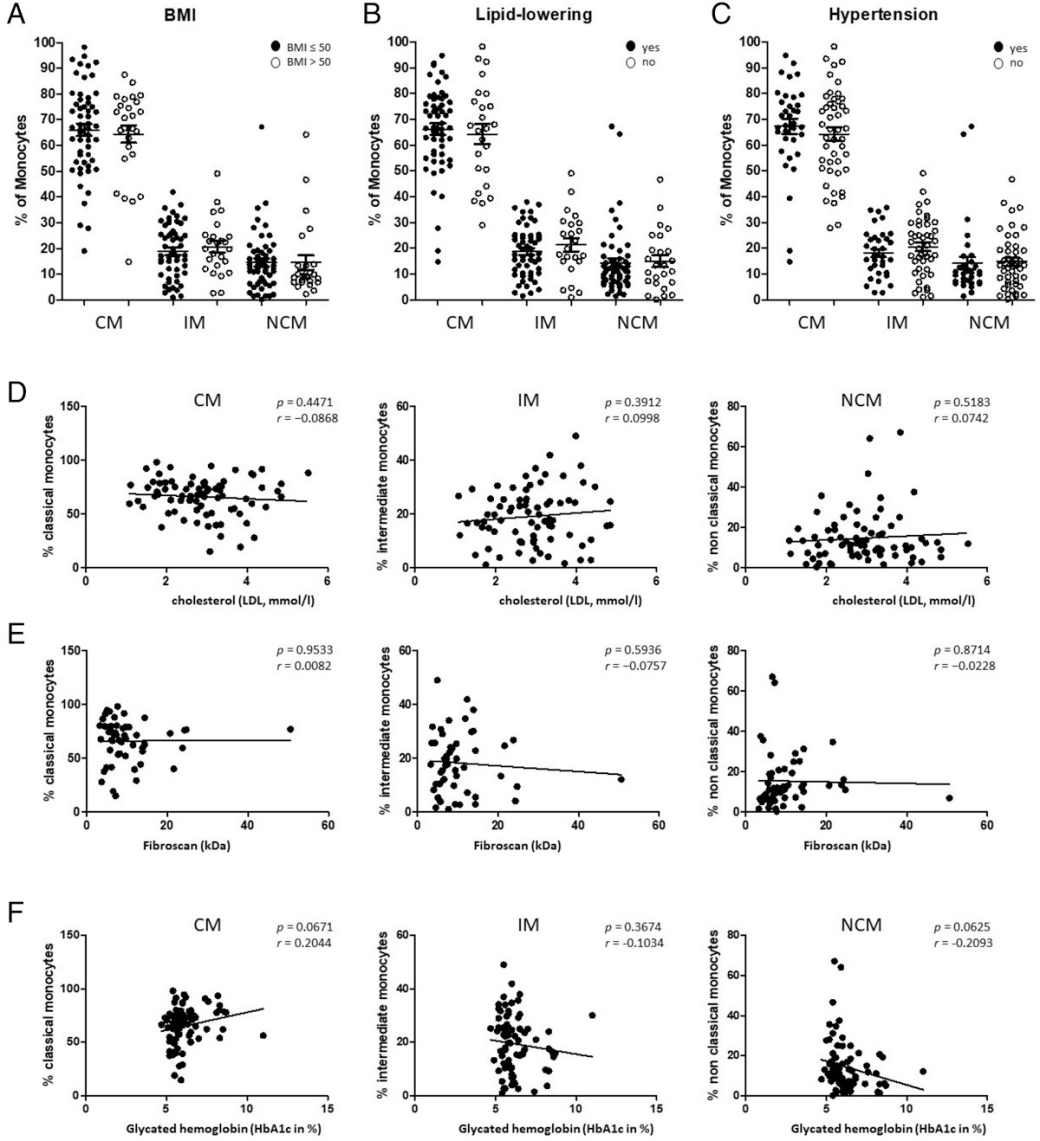
Flow cytometric analysis of subcohorts of patients with obesity. (**A**) Cohort subdivision in terms of BMI values (≤50, *n* = 57 versus >50, *n* = 25) revealed no significant differences. (**B**) Cohort subdivision in terms of lipid-lowering medications (yes, *n* = 25 versus no, *n* = 57) revealed no significant differences. (**C**) Cohort subdivision in terms of hypertension (yes, *n* = 46 versus no, *n* = 36) revealed no significant differences. (**D**) Correlation analyses between monocyte subset percentages and measured cholesterol values (low-density lipoprotein [LDL], mmol/l) revealed no significant differences. (**E**) Correlation analyses between monocyte subset percentages and measured liver fibrosis (FibroScan, kDa) values revealed no significant differences. (**F**) Correlation analyses between monocyte subset percentages and HbA1c values revealed no significant differences.

Gating of CD14/CD16-characterized monocyte subgroups was performed as previously described (13). In summary, CD45 was used as a pan leukocyte marker to facilitate whole blood measurement, and monocytes were first roughly gated by their Forward Scatter/Side Scatter characteristics and the positivity for CD14 and CD16. Neutrophil granulocytes and NK cells were excluded by their missing HLA-DR expression. Remaining B cells were excluded by the help of their lack of CD14 expression. Finally, the remaining monocytes were subgated into CD14^2+^CD16^−^ (classical), CD14^2+^CD16^+^ (intermediate), and CD14^dim+^CD16^+^ (nonclassical) monocytes (Fig. 1A). We identified significantly decreased abundances of CMs in obesity patients accompanied by significantly increased percentages of the IM and NCM subsets (Fig. 1B). Due to the strongly heterogeneous distributions of the abundances of the different monocyte subsets in the analyzed patients with obesity, the cohort was further subdivided into a lower and higher group in terms of BMI values (≤50 versus >50), lipid-lowering medications, and hypertension, respectively, independently of additional comorbidities. Our data revealed again heterogeneous distributions of the abundances of the different monocyte subsets, but no significant differences between the defined subgroups (Fig. 2A–C). Further correlations between the percentages of CM, IM, and NCMs with the measured cholesterol, liver fibrosis (FibroScan), and HbA1c values revealed as well no significant differences (Fig. 2D–F).

**FIGURE 3.**
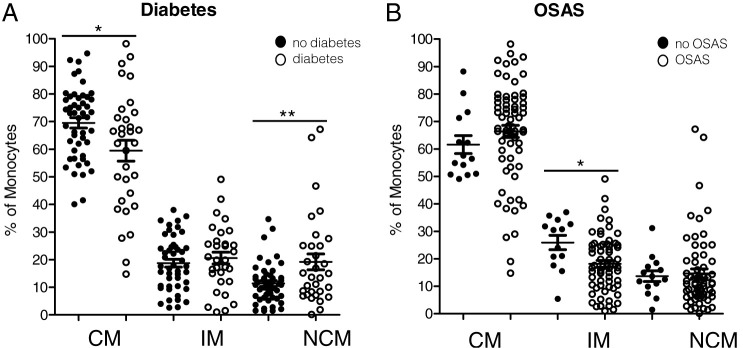
Flow cytometric analyses of diabetes and OSAS-related monocyte subset abundances in patients with obesity. (**A**) Whole blood analysis revealed significantly decreased percentages of CMs accompanied by significantly increased percentages of NCMs in diabetes-positive patients with obesity compared with the nondiabetes group. (**B**) Cohort subdivision in terms of OSAS revealed significantly decreased percentages of IMs in OSAS-positive patients compared with the OSAS-negative group. *p < 0.05, **p < 0.01.

Next, the impact of diabetes mellitus on the distribution of peripheral blood monocyte subsets was investigated. Our data revealed significantly decreased percentages of CMs (*p* = 0.0105) and significantly increased percentages of NCMs (*p* = 0.0047) in patients with diabetes compared with patients without diabetes (Fig. 3A). Moreover, patients with obesity were stratified by status of OSAS, diagnosed by polysomnography and recordings of snoring and heart rate by a portable device. Analyses of monocyte subset abundances revealed no significant differences of CMs and NCMs in patients with versus without OSAS, but significantly decreased percentages of IMs (*p* = 0.0143) in patients with obesity and OSAS (Fig. 3B).

**FIGURE 4.**
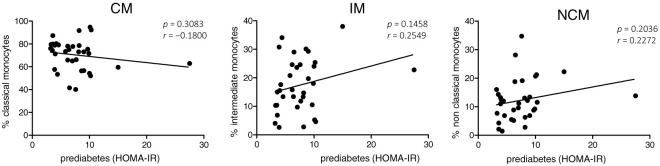
Correlation analysis between the percentages of CMs, IMs, and NCMs and the HOMA-IR values of prediabetes patients revealed a correlation by tendency. The Pearson correlation coefficient (*r*) and *p* values are given. A *p* value <0.05 was considered significant.

Based on the homeostasis model assessment-insulin resistance (HOMA-IR), we further stratified patients without diabetes into patients with prediabetes and patients with physiological glucose homeostasis. Correlation analyses between the percentages of the three monocyte subsets and the achieved HOMA-IR values revealed a corresponding influence by tendency, thus corroborating the impact of diabetes (Fig. 4). These data indicate that obesity is associated with strong redistribution of circulating monocyte subsets hardly affected by concomitant OSAS, whereas diabetes even amplifies the observed alterations.

### Obesity-related monocytic adhesion molecules

**FIGURE 5.**
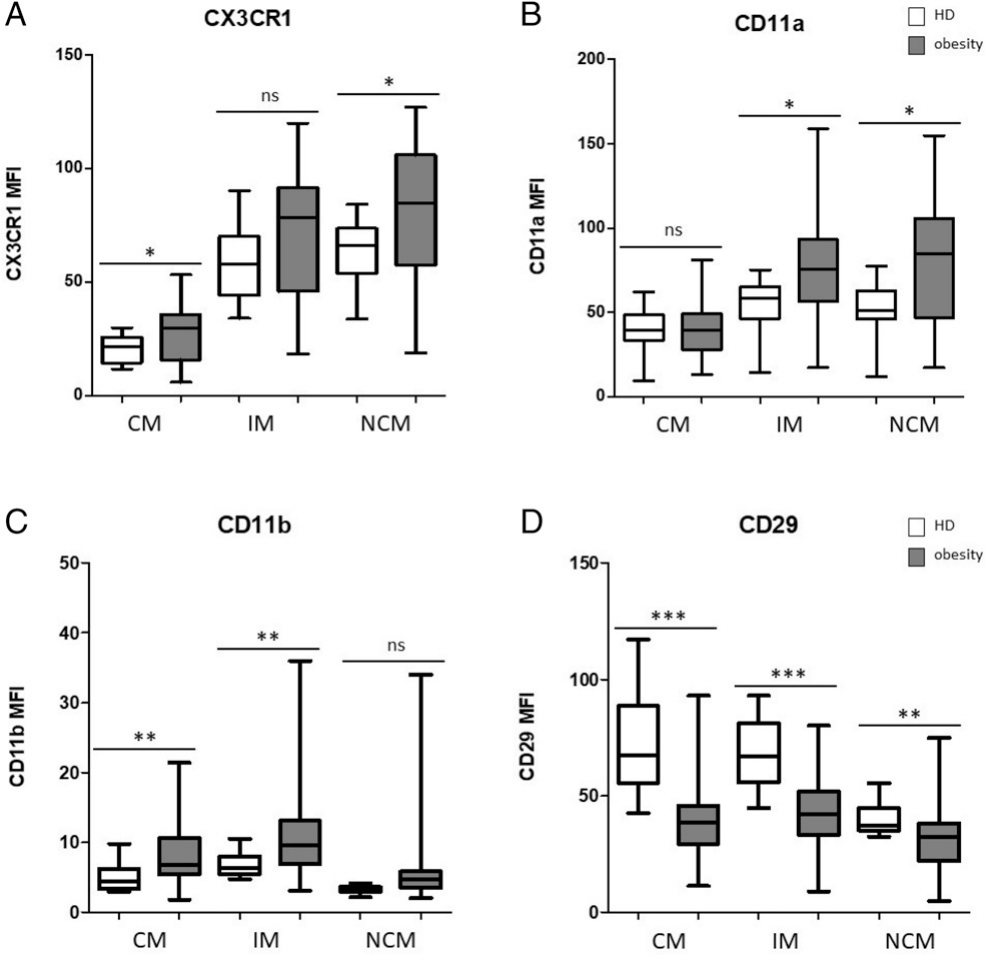
Flow cytometric analyses of monocytic adhesion molecules in patients with obesity compared with healthy donors. (**A**) The expression of CX3CR1 was significantly higher on CMs and NCMs in patients with obesity compared with healthy donors and higher on the IMs and NCMs compared with CMs. (**B**) The expression of CD11a was significantly higher on IMs and NCMs from patients with obesity compared with healthy donors and higher on IMs and NCMs compared with CMs. (**C**) The expression of CD11b was significantly higher on CMs and IMs in patients with obesity compared with healthy donors and revealed strongly heterogeneous distributions among the analyzed patient cohort. (**D**) The expression of CD29 was significantly lower on CMs, IMs, and NCMs in patients with obesity compared with healthy donors. *p < 0.05, **p < 0.01, ***p < 0.001. ns, not significant.

In addition, expression levels of adhesion molecules and chemokine receptors CD11a (integrin-α L; LFA-1), CD11b (integrin-α M; Mac-1), CD11c (integrin-α X), CD18 (integrin β-2), CD29 (integrin β-1), CD49d (integrin β-4), CD162 (P-selectin receptor), and CX3CR1 (CX3CL1 receptor) were analyzed in patients with obesity and compared with healthy control subjects using flow cytometry. As a whole, the expression levels of CD11c, CD18, CD49d, and CD162 were highly heterogeneous on circulating monocytes from patients with obesity as compared with healthy control subjects, but they revealed no significant differences (data not shown). The expression of CX3CR1 was significantly higher on CMs (*p* = 0.0205) and NCMs (*p* = 0.0223) and higher on the IMs and NCMs compared with CMs (Fig. 5A). The expression of CD11a was significantly higher on IMs (*p* = 0.0145) and NCMs (*p* = 0.0052) and also higher on the IMs and NCMs compared with CMs (Fig. 5B). The expression of CD11b was significantly higher on CMs (*p* = 0.0064) and IMs (*p* = 0.0065) in patients with obesity compared with healthy control subjects and revealed quite heterogeneous distributions within the patient cohort (Fig. 5C). The expression of CD29 was significantly lower on CMs (*p* < 0.001), IMs (*p* < 0.001), and NCMs (*p* = 0.0054) in patients with obesity compared with healthy controls (Fig. 5D).

### Impact of obesity-related plasma adipokines

Obesity is accompanied by changed levels of different circulating adipokines, which are related to the development of obesity-associated diseases. Therefore, we analyzed the plasma levels of adipokines adiponectin and leptin using ELISA measurements. Our data revealed heterogeneous plasma levels of adiponectin and leptin in patients with obesity without significant differences between patients with versus without OSAS. Further correlation analyses revealed no significant correlations between the measured adiponectin plasma concentrations and all other clinical parameters or the observed monocyte subset alterations (data not shown).

**FIGURE 6.**
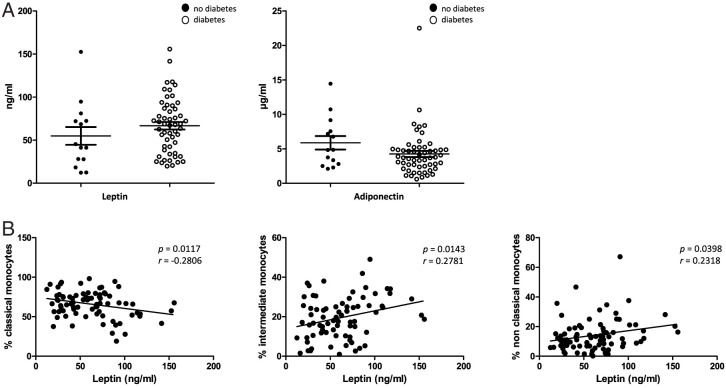
ELISA measurements of plasma adipokines. (**A**) Measurements of adipokines leptin and adiponectin revealed heterogeneous plasma levels in the analyzed cohort but no significant differences between OSAS-positive and OSAS-negative patients with obesity. (**B**) Correlation analyses revealed significant correlations between the percentages of CMs (*p* = 0.0117), IMs (*p* = 0.0143), and NCMs (*p* = 0.0398) and the measured plasma leptin levels. The Pearson correlation coefficient (*r*) and *p* values are given. A *p* value <0.05 was considered significant.

However, correlation analyses revealed significant correlations between the measured plasma leptin levels and percentages of CMs (*p* = 0.0117), IMs (*p* = 0.0143), and NCMs (*p* = 0.0398) (Fig. 6B).

## Discussion

### Distribution of circulating monocyte subsets

Characterizing the impact and interplay of obesity and its concomitant diseases on the specifics of peripheral blood monocyte subsets, these data reveal highly significant redistributions of the three CD14/CD16 monocyte subsets and clinical characteristics as, e.g., BMI, lipid-lowering medications, hypertension, HbA1c, lipids, or liver fibrosis.

Earlier studies observed a positive correlation between the individual BMI and the percentage of resident macrophages, suggesting that fat tissue growth is associated with an increased recruitment of circulating monocytes (21). Indeed, we have recently shown a positive correlation between the redistribution of circulating monocyte subsets and the BMI in patients with OSAS (13). The fact that we observed no significant correlations between CM and NCM subset percentages and individual BMI values in this study cohort is most likely due to the significantly higher median BMI of 46.8 compared with 29.2 in the previous OSAS cohort, which strongly suggests that all individuals of the present cohort exceeded the BMI limit responsible for persisting systemic inflammation.

Next, our data revealed significantly decreased percentages of CMs and significantly increased abundances of CD16^+^ NCMs in patients with obesity and concomitant diabetes. Decreased percentages of CD14^+^ CMs and increased abundances of IMs have also been observed in gestational diabetes mellitus, in which a hormone (human chorionic gonadotropin) made by the placenta prevents the body from using insulin effectively (22). These data underline the relevant impact of glucose homeostasis on monocyte subset abundances in patients with obesity additive factors of immune disturbance.

### Alteration of monocytic adhesion molecules

Monocyte adhesion to endothelial cells in the blood vessels and their transmigration is mediated by various adhesion molecules and chemokine receptors. CMs are considered to have greater potential to migrate into sites of inflammation than IMs or NCMs (23). Our data revealed increased expression levels of CD11a (integrin-α L; LFA-1), CD11b (integrin-α M; Mac-1), and CX3CR1 (CX3CL1 receptor) and decreased expression of CD29 (integrin β-1) on certain monocyte subsets in patients with obesity.

As leukocyte adhesion molecules, integrins CD11a and CD11b mediate immune cell binding to endothelial cells and belong to the characteristic signature of inflammatory monocytes in patients with coronary artery diseases (24). However, CD11b has also been shown to act as a suppressor of immune responses in myeloid cells (25–27). Adhesion molecule CX3CR1 is required for monocyte crawling along the blood vessels by mediating the interaction with the endothelium (28). Both human and mouse CD16^+^ monocyte subsets have been shown to express high levels of the chemokine receptor CX3CR1 and to respond to CX3C-chemokine ligand 1 (28). Correspondingly, CX3CR1 is also associated with atherosclerosis and vascular inflammatory processes (29, 30). However, only little is known about CX3CR1 in terms of the differentiation cascades of monocyte subsets and macrophages (31).

The differential contribution of monocyte subsets and their chemokine receptor expression pattern to atherosclerosis is still unclear. In this context, it has recently been shown that the impact of CCR2^+^ monocyte subpopulations even reveals sexual dimorphism, where boys seem to be at higher atherosclerosis risk than girls (32). Further, it has been suggested that proinflammatory circulating monocytes are particularly involved in the early stages of atherosclerosis development (33).

The β_1_ integrin CD29 plays an important role in the regulation of cell attachment and migration of immune cells in inflammatory processes (34). Although the molecular regulation of CD29 has not been fully elucidated yet, it has been suggested that it might be modulated by its regulatory adhesion molecules CD98 (a cell-surface glycoprotein) and CD147 (basigin; glycoprotein coreceptor) via the actin cytoskeleton (35). Furthermore, it has recently been shown that inhibition of the integrin β1/PYK2 axis has a negative impact on the transendothelial migration of monocytes, as well as the cancer-promoting functions of tumor-associated macrophages (36).

### Influence of plasma adipokines

Obesity is accompanied by alterations in the circulating levels of several adipokines, which are responsible for an underlying chronic state of low-grade inflammation and therefore contribute to the development of concomitant diseases, such as type 2 diabetes, hypertension, atherosclerosis, and some types of cancer (37–39).

Our data revealed heterogeneous plasma levels of adiponectin within our patient cohort. In terms of adiponectin, there were no significant differences between OSAS-positive and OSAS-negative patients. These data corroborate earlier studies that observed significantly higher plasma TNF-α levels in OSAS patients compared with healthy controls (40). Further correlation analyses revealed no significant correlations between the measured adiponectin plasma concentrations and the clinical phenotype or the identified monocyte subset alterations.

In contrast, our data revealed a significant negative correlation between the plasma leptin levels and percentages of CMs, as well as significant positive correlations with the abundances of IMs and NCMs. Leptin is a common adipokine that is secreted by white adipose tissue and is involved in the regulation of the blood glucose metabolism and energy expenditure (41, 42). Our data revealed no significant differences of the plasma leptin levels between OSAS-positive and OSAS-negative patients, most likely because of the high median BMI of our study cohort. The specific influence of obesity and OSAS on individual plasma leptin levels is controversially discussed. Some studies suggested that OSAS patients have higher plasma levels of leptin (43), whereas others observed no differences in serum leptin between OSAS patients and controls (44). Further, there is strong evidence that leptin is also involved in the regulation of cell-mediated inflammatory processes. It has been shown that peripheral leptin treatment leads to increased numbers of granulocytes, NK cells, monocytes, and hematopoietic progenitors (45, 46). Our data revealed a relevant impact of obesity per se on the distribution and characteristics of peripheral blood monocyte subsets, with plasma leptin levels, OSAS, and diabetes as crucial amplifying factors. Further analyses of immune functions and total counts of distinct monocyte subsets and ongoing comprehensive investigations on larger patient cohorts in terms of OSAS and diabetes treatment and weight reduction over a longer period will help to better understand the multifactorial regulation of the peripheral immunity in obesity.
